# Gut microbiota lipopolysaccharide accelerates inflamm-aging in mice

**DOI:** 10.1186/s12866-016-0625-7

**Published:** 2016-01-16

**Authors:** Kyung-Ah Kim, Jin-Ju Jeong, Sul-Young Yoo, Dong-Hyun Kim

**Affiliations:** Department of Life and Nanopharmaceutical Sciences, College of Pharmacy, Kyung Hee University, 1, Hoegi, Dongdaemun-ku, Seoul 130-701 Korea; Department of Food and Nutrition, Song Won University, 73, Songamro, Nam-gu, Gwangju 503-742 Korea

**Keywords:** Inflamm-aging, Gut microbiota, Lipopolysaccharide, p16, SAMHD1

## Abstract

**Background:**

The constitutive inflammation that characterizes advanced age is termed inflamm-aging. This process is associated with age-related changes to immune homeostasis and gut microbiota. We investigated the relationship between aging and gut microbiota lipopolysaccharide (LPS)-inducible inflammation.

**Results:**

A taxonomy-based analysis showed that aging resulted in increased prevalence of the phyla *Firmicutes* and *Actinobacteria* and a reduced prevalence of *Bacteroidetes* and *Tenericutes*, resulting in an increase in the *Firmicutes* to *Bacteroidetes* ratio. The levels of plasmatic and fecal lipopolysaccharides were higher in aged mice. Aging induced the expression of p16 and the activation of nuclear factor-kappa B (NF-κB) in the colon of aged mice. Interestingly, the expression level of sterile α-motif domain- and HD domain-containing protein 1 (SAMHD1) in the colon was higher in aged mice than in young mice, while cyclin-dependent kinase-2 and cyclin E levels were lower in aged mice than in young mice. The lipopolysaccharide fraction of fecal lysates (LFL) from young or aged mice increased p16 and SAMHD1 expression and NF-κB activation in peritoneal macrophages from wild-type mice, in a TLR4-dependent manner. However, LFLs did not induce NF-κB activation and SAMHD1 expression in peritoneal macrophages from TLR4-deificent mice, whereas they significantly induced p16 expression. Nevertheless, p16 expression was induced more potently in macrophages from WT mice than in macrophages from TLR4-deficient mice.

**Conclusion:**

Aging increased p16 and SAMHD1 expression, gut microbiota LPS production, and NF-κB activation; thereby, signifying that gut microbiota LPS may accelerate inflamm-aging and SAMHD1 may be an inflamm-aging marker.

## Background

Aging is a degenerative process that is strongly associated with inflammation. Chronic low-grade inflammation typical of aging, termed “inflamm-aging”, involves inflammatory network activation and the release of senescence-associated factors, including key pro-inflammatory mediators, such as nuclear factor-kappa B (NF-κB) [1, 2]. Inflamm-aging may also be associated with age-related changes to oxidative/genotoxic stress and gut microbiota composition [3].

In addition to gastrointestinal disease, metabolic disorders, and antibiotic use, aging has also been shown to affect the gut microbiota composition; the levels of gut *Bifidobacteria* decline with age, whereas those of *Clostridium perfringens*, *Lactobacilli*, and *Enterococci* increase with age [4]. This may be salient in the context of inflammation since the composition of the gut microbiota has been shown to strongly correlate with intestinal inflammatory diseases [5]. Despite these observations, however, the mechanism through which the gut microbiota composition induces low-grade inflammation at the molecular level remains unclear.

Cell-cycle regulators have long been considered to play important roles in the induction of senescence in cultured cells. Among these molecules, p16 has recently been singled out as a suitable marker of senescence in vivo [6]. Senescence is induced by p16 through inhibition of the activity of the cyclin-dependent kinases CDK4 and CDK6, which would otherwise phosphorylate and inactivate the retinoblastoma tumor suppressor. With age, the expression of p16 increases in the stem and progenitor cells of mice and suppresses stem cell proliferation and tissue regeneration [7–9].

Recent studies have shown that sterile α-motif domain- and HD domain-containing protein 1 (SAMHD1), a cellular deoxynucleoside triphosphohydrolase related to cell replication, prevents viral replication by depleting the cellular deoxynucleoside triphosphate pool available for reverse transcription of viral DNA [10, 11]. SAMHD1, which is highly expressed in non-dividing cells such as macrophages and dendritic cells [12], regulates cell proliferation by cyclin A2/CDK1 [13]. Furthermore, SAMHD1 is suggested to regulate the cell cycle by the degradation of cellular dNTP. However, little is known about the functional role of SAMHD1 within cells.

In this study, we first investigated the composition and LPS production levels of gut microbiota and protein expression levels of inflamm-aging markers such as p16, NF-κB, and SAMHD1 in young and aged mice and the role of aging. Furthermore, we investigated the relationship between aging and gut microbiota LPS-induced inflammation.

## Methods

### Animals and diets

All experiments were performed in accordance with the NIH and Kyung Hee University guidelines for Laboratory Animals Care and Use and approved by the Committee for the Care and Use of Laboratory Animals at the College of Pharmacy, Kyung Hee University (KHP-2012-04-1).

Male C57BL/6J mice (4 or 18 months old) and TLR4-deficient C57BL/10ScNJ mice (4 months old) were purchased from Jackson Laboratory (Bar Harbor, ME, USA). Each group consisted of eight mice. All mice were housed in wire cages at 20–22 °C and 50 % ± 10 % humidity and fed 10 kcal % fat diet (D12450B) obtained from Research Diets, Inc. (New Brunswick, NJ, USA) for 8 weeks. For biochemical assays, mice were then anesthetized, and blood samples were collected. The colon was quickly removed, opened longitudinally, gently cleared of stool using phosphate-buffered saline (PBS), and used for ELISA and immunoblotting.

### DNA extraction, pyrosequencing, and data analysis

Genomic DNA was extracted from four fecal samples of each group using a commercial DNA isolation kit (QIAamp DNA Stool Mini Kit, Qiagen, Hilden, Germany) following the manufacturer’s protocol. Amplification of genomic DNA was performed using barcoded primers that targeted the V1 to V3 regions of the bacterial 16S rRNA gene. The sequencing and basic analysis were performed according to methods described by Chun et al*.* [14] and completed by ChunLab Inc. (Seoul, Korea) by using a 454 GS FLX Titanium Sequencing System (Roche, Branford, CT, USA). Sequences for each sample were sorted by a unique barcode and low quality reads (average quality score <25 or read length <300 bp) were removed. Sequence reads were identified using the EzTaxon-e database (http://eztaxon-e.ezbiocloud.net/) on the basis of 16S rRNA sequence data [15]. The number of sequences analyzed, observed diversity richness (OTU), estimated OTU richness (ACE and Chao1), and pyrosequencing coverage were calculated using the Mothur program and defined considering a cut-off value of 97 % similarity of the 16S rRNA gene sequences. The distances between microbial communities from each sample were represented as an Unweighted Pair Group Method with Arithmetic Mean (UPGMA) clustering tree describing the dissimilarity between multiple samples. 454 Pyrosquencing reads were deposited in NCBI’s short read archive (accession number SRP056060).

### Limulus amebocyte lysate assay

Plasma and fecal endotoxin contents were determined using diazo-coupled limulus amoebocyte lysate (LAL) assays (Cape Cod Inc., E. Falmouth, MA, USA) according to the manufacturer’s protocol. Briefly, plasma was diluted 1:10 in pyrogen free water, inactivated for 10 min at 70 °C and then incubated with LAL for 30 min at 37 °C. Addition of reagents led to formation of a magenta derivative that absorbed light at 545 nm. For the fecal endotoxin assay, 20 mg of feces from the cecum was placed in 50 mL PBS in a pyrogen-free tube and sonicated for 1 h on ice. After centrifugation at 400 × *g* for 15 min, the upper 30 mL was collected, sterilized by filtration through a 0.45-μm filter, re-filtered through a 0.22-μm filter, and inactivated for 10 min at 70 °C. The LPS content of the filtered sonicate (LPS fraction of the fecal lysate, LFL) was then assayed using LAL assay kit.

### ELISA and immunoblotting

The concentrations of tumor necrosis factor alpha (TNFα), interleukin (IL)-1β, and IL-6 in the plasma and colon were determined using commercial ELISA kits (R&D Systems, Minneapolis, MN, USA). The levels of p16, beclin-1, ATG7, LC3, phosphorylated mammalian target of rapamycin (mTOR), mTOR, phosphorylated p65, p65, SAMHD1, cyclin E, CDK2, and β-actin proteins in the colon and collected cells were assayed as previously described [16]. Immunodetection was performed using an ECL detection kit (Pierce Co., Rockford, IL, USA).

### Isolation and culture of peritoneal macrophages

Peritoneal macrophages from male C57BL/6J mice or TLR4-deficient mice were isolated as described previously [16]. Mice were injected intraperitoneally (i.p.) with 2 mL of 4 % thioglycolate solution. Mice were killed 4 days after injection and the peritoneal cavities were washed with 10 mL of Roswell Park Memorial Institute (RPMI) 1640 medium. The peritoneal lavage fluids were centrifuged at 200 × g for 10 min and the cells were RPMI 1640 medium and plated. After incubation for 1 h at 37 °C, the cells were washed three times and non-adherent cells were removed by aspiration. The attached cells were used as peritoneal macrophages were cultured in 24-well plates (0.5 × 10^6^ cells/well) at 37 °C in RPMI 1640 containing 10 % fetal bovine serum.

To examine the inflammatory effects of fecal lysates of mice, peritoneal macrophages were incubated with 100 μL of fecal lysates and used for immunoblotting. LPS purchased from Sigma-Aldrich (St. Louis, MO, USA) was used as standard.

### Statistical analysis

The data are expressed as the means ± standard errors of the means. Statistical analysis of the data was performed with one-way analysis of variance (ANOVA). Differences with a *p* value of less than 0.05 were considered statistically significant.

## Results

### Gut microbial communities in young and aged mice

We began our investigation by examining the gut microbiota from mice at different ages. Cecal samples were collected from mice maintained for 8 weeks on a normal (low-fat) diet to investigate changes in the gut microbiota by pyrosequencing. The number of sequences analyzed, OTUs, estimated OTU richness (ACE and Chao1), and pyrosequencing coverage were calculated using Cluster Database at High Identity with Tolerance (Table 1). Decreased bacterial richness and diversity were observed in cecal samples collected from aged mice relative to those from young mice; however, these differences are not statistically significant.

**Table 1:** Number of sequences analyzed, observed diversity richness (OTU), estimated OTU richness (ACE and Chao1), and coverage

	Total reads	OTUs	ACE	Chao1	Coverage
YM-1	3826	423	822.8	673.3	0.95
YM -2	3427	448	994.3	744.1	0.94
YM -3	3015	435	1058.9	781.6	0.93
YM -4	3751	449	824.6	683.4	0.95
Mean ± SEM	3504.8 ± 369.6	438.8 ± 12.3	925.2 ± 120.1	720.6 ± 51.3	0.94 ± 0.01
AM -1	4066	479	996.8	745.5	0.95
AM -2	3619	385	709.9	580.7	0.96
AM -3	3772	387	731.0	681.1	0.96
AM -4	3786	374	772.3	640.1	0.96
Mean ± SEM	3810.8 ± 186.2	406.3 ± 48.8	802.5 ± 132.1	661.9 ± 69.4	0.95 ± 0.01

All values are indicated as the mean ± standard error of the mean

*YM* young mice; *AM* aged mice

**Fig. 1:**
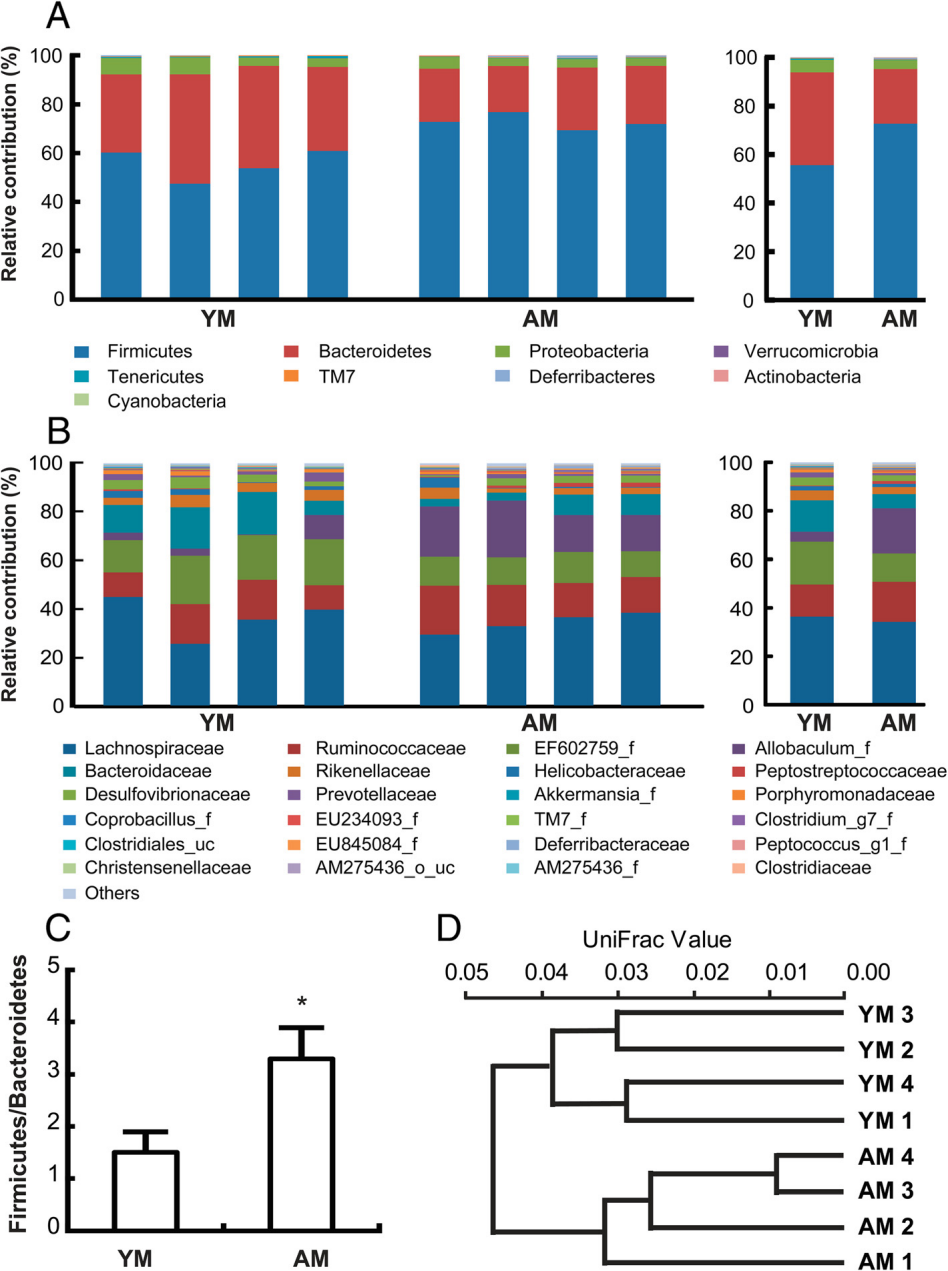
The composition of intestinal microbiota. The relative contributions of dominant (**a**) phyla and (**b**) families (individual samples are shown in the left panels and pooled samples are shown in the right panels) and (**c**) the *Firmicutes* to *Bacteroidetes* ratio are shown as identified from pyrosequencing data. **d** Hierarchical clustering of gut microbial gene expression profiles. The distances between microbial communities from each sample are represented as an Unweighted Pair Group Method with Arithmetic Mean (UPGMA) clustering tree describing the dissimilarity between multiple samples. All values are indicated as the mean ± standard error of the mean (*n* = 4). *, *p* < 0.05 in comparison with young mice. YM, young mice; AM, aged mice

Taxonomy-based analysis showed that aging significantly modulated the populations of the dominant intestinal microbiota. At the phylum level, decreased levels of *Bifidobacteria*, which have been shown to exhibit anti-inflammatory effects in mice, and increased levels of *Lactobacilli* and *Enterococci* have been reported in elderly individuals compared with young adults [4]. In the present study, aging increased the prevalence of *Firmicutes* (*p* = 0.002) and *Actinobacteria* (*p* = 0.034) and reduced the prevalence of *Bacteroidetes* (*p* = 0.003) and *Tenericutes* (*p* = 0.038) (Fig. 1a,b), leading to an overall increase in the *Firmicutes* to *Bacteroidetes* ratio (*p* = 0.002) in the gut microbiota (Fig. 1c).

At the family level, *Allobaculum_f* (*p* = 0.002) and *Clostridiaceae* (*p* = 0.039) populations decreased, while *Clostridiales_uc* (*p* = 0.04) and EF602759_f (*p* = 0.008, phylum *Bacteroidetes*) populations were enriched in aged mice compared with young mice (Fig. 1b).

At the genus level, there were seven predominant genera. Six genera were in the phylum *Firmicutes* and one genus was in the phylum *Bacteroidetes*: DQ789121_g (phylum *Firmicutes*), *Pseudoflavonifractor*, EF603943_g (phylum *Firmicutes*), *Bacteroides*, EF603662_g (phylum *Firmicutes*), AB626958_g (phylum *Firmicutes*), and *Clostridium_g9*. Collectively, these seven genera accounted for more than 50 % of the sequences (Table 2). Among the 25 most abundant genera, EF603943_g, *Clostridium_g9*, and DQ815556_g of the phylum *Firmicutes* were enriched, while AY239469_g and EF406712_g of the phylum *Bacteroidetes* were reduced in aged mice in comparison with young mice.

**Table 2:** Difference in the composition (percent of total sequences) of fecal bacterial genera isolated from young mice and aged mice

	Composition^*^ (%)	
Genus	YM	AM
*DQ789121_g (Clostridiales)*	1.64 ± 1.95	0.72 ± 1.05
*Pseudoflavonifractor*	5.92 ± 1.79	7.47 ± 8.92
*EF603943_g (Allobaculum)*	4.03 ± 4.16	17.68 ± 3.91^*^
*Bacteroides*	12.96 ± 5.48	5.88 ± 3.07
*EF603662_g (Clostridiales)*	15.36 ± 12.73	5.86 ± 5.30
*AB626958_g (Clostridiales)*	7.66 ± 6.59	10.49 ± 8.53
*Clostridium_g9*	2.50 ± 1.52	5.16 ± 0.52^*^
*Oscillibacter*	3.60 ± 1.65	5.64 ± 1.59
*Helicobacter*	1.74 ± 1.11	1.30 ± 1.94
*HM124280_g (Bacteroidales)*	4.31 ± 2.49	3.40 ± 1.49
*Clostridium_g4*	0.27 ± 0.31	1.17 ± 0.67
*Alistipes*	3.59 ± 1.03	2.89 ± 1.25
*EF602759_g (Bacteroidales)*	2.31 ± 1.05	1.93 ± 0.14
*AY239469_g (Bacteroidales)*	3.59 ± 0.94	1.85 ± 1.05^*^
*DQ815907_g (Desulfovibrionales)*	3.26 ± 1.20	2.23 ± 1.37
*Alloprevotella*	2.01 ± 1.31	0.94 ± 0.56
*EF406806_g (Bacteroidales)*	1.30 ± 0.82	1.89 ± 0.54
*EF406712_g (Bacteroidales)*	2.64 ± 0.58	0.87 ± 0.19^*^
*DQ815556_g (Clostridiales)*	0.25 ± 0.10	1.38 ± 0.75^*^
*Lachnospiraceae_uc*	1.70 ± 0.52	0.96 ± 0.52
*DQ789117_g (Clostridiales)*	0.23 ± 0.16	1.69 ± 0.52
*Acetatifactor*	0.24 ± 0.13	1.44 ± 0.46^*^
*Akkermansia*	0.01 ± 0.03	nd
*AB626943_g (Clostridiales)*	2.25 ± 3.16	0.15 ± 0.19
*EU006213_g (Clostridiales)*	0.08 ± 0.10	0.46 ± 0.91

All values are indicated as the mean ± standard error of the mean

*YM* young mice; *AM* aged mice, *nd* not detectable

^*^ *p* < 0.05 in comparison with young mice

We also analyzed the differences in the gut microbiota composition at the species level (Table 3). EF603943_s (*Erysipelotrichales*), FJ881142_s (*Clostridiales*), AY991787_s (*Clostridiales*), DQ815350_s (*Clostridiales*), EF098042_s (*Clostridiales*), AB606316_s AB606316_s (*Clostridiales*) of the phylum *Firmicutes* were enriched, while EF406712_s (*Bacteroidales*) and EF406368_s (*Bacteroidales*) of the phylum *Bacteroidetes* were reduced in aged mice in comparison with young mice.

**Table 3:** Difference in the composition (percent of total sequences) of fecal bacterial species isolated from young mice and aged mice

	Composition (%)	
Species	YM	AM
*EU510908_s (Clostridiales)*	0.20 ± 0.28	0.05 ± 0.04
*EF603943_s (Erysipelotrichales)*	4.01 ± 4.14	17.59 ± 3.91^*^
*EF603662_s (Clostridiales)*	15.25 ± 12.68	5.83 ± 5.26
*DQ789121_s (Clostridiales)*	0.07 ± 0.08	0.04 ± 0.03
*AB599946_s (Bacteroidales)*	6.81 ± 4.60	4.85 ± 3.75
*EU504374_s (Clostridiales)*	0.07 ± 0.10	8.88 ± 8.54
*FJ881142_s (Clostridiales)*	0.27 ± 0.30	1.11 ± 0.61^*^
*GQ157662_s (Bacteroidales)*	4.13 ± 2.53	3.14 ± 1.56
*AB626958_g_uc (Clostridiales)*	5.97 ± 5.16	0.38 ± 0.27
*EU504931_s (Clostridiales)*	1.03 ± 0.29	1.13 ± 0.14
*Helicobacter hepaticus*	0.01 ± 0.01	1.30 ± 1.94
*EF097240_s (Bacteroidales)*	2.00 ± 1.31	0.93 ± 0.56
*EF096080_s (Desulfovibrionales)*	3.21 ± 1.19	2.18 ± 1.46
*EF406368_s (Bacteroidales)*	3.52 ± 0.95	1.63 ± 1.14^*^
*AF157051_s (Clostridiales)*	0.56 ± 0.47	1.92 ± 1.04
*Bacteroides acidifaciens*	1.24 ± 1.01	0.84 ± 0.95
*Bacteroides vulgatus*	4.35 ± 3.59	nd
*EF406712_s (Bacteroidales)*	2.56 ± 0.52	0.85 ± 0.19^*^
*AY991787_s (Clostridiales)*	0.07 ± 0.04	0.73 ± 0.16^*^
*Lachnospiraceae_uc_s*	1.70 ± 0.52	0.96 ± 0.52
*EF603688_s (Bacteroidales)*	1.94 ± 0.83	1.13 ± 1.43
*JQ083832_s (Clostridiales)*	0.52 ± 0.41	0.76 ± 0.27
*DQ815350_s (Clostridiales)*	0.03 ± 0.04	1.58 ± 0.57^*^
*EF098042_s (Clostridiales)*	0.07 ± 0.13	0.91 ± 0.56^*^
*AB606316_s (Clostridiales)*	0.13 ± 0.15	1.56 ± 0.57^*^

All values are indicated as the mean ± standard error of the mean

*YM* young mice; *AM* aged mice, *nd* not detectable

^*^ *p* < 0.05 in comparison with young mice

As shown in Fig. 1d, the samples from different groups could be readily separated, and four samples from each group clustered together.

### Plasma and fecal endotoxin levels in young and aged mice

Inflammatory markers such as CD14, which is a LPS-binding protein in TLR4-linkged NF-κB signaling pathway, and C-reactive protein increase with advancing ages [17]. A previously unconsidered source of inflammatory initiation is the translocation of gut microbiota and their products, leading to inflammation such as inflammatory bowel disease.

**Fig. 2:**
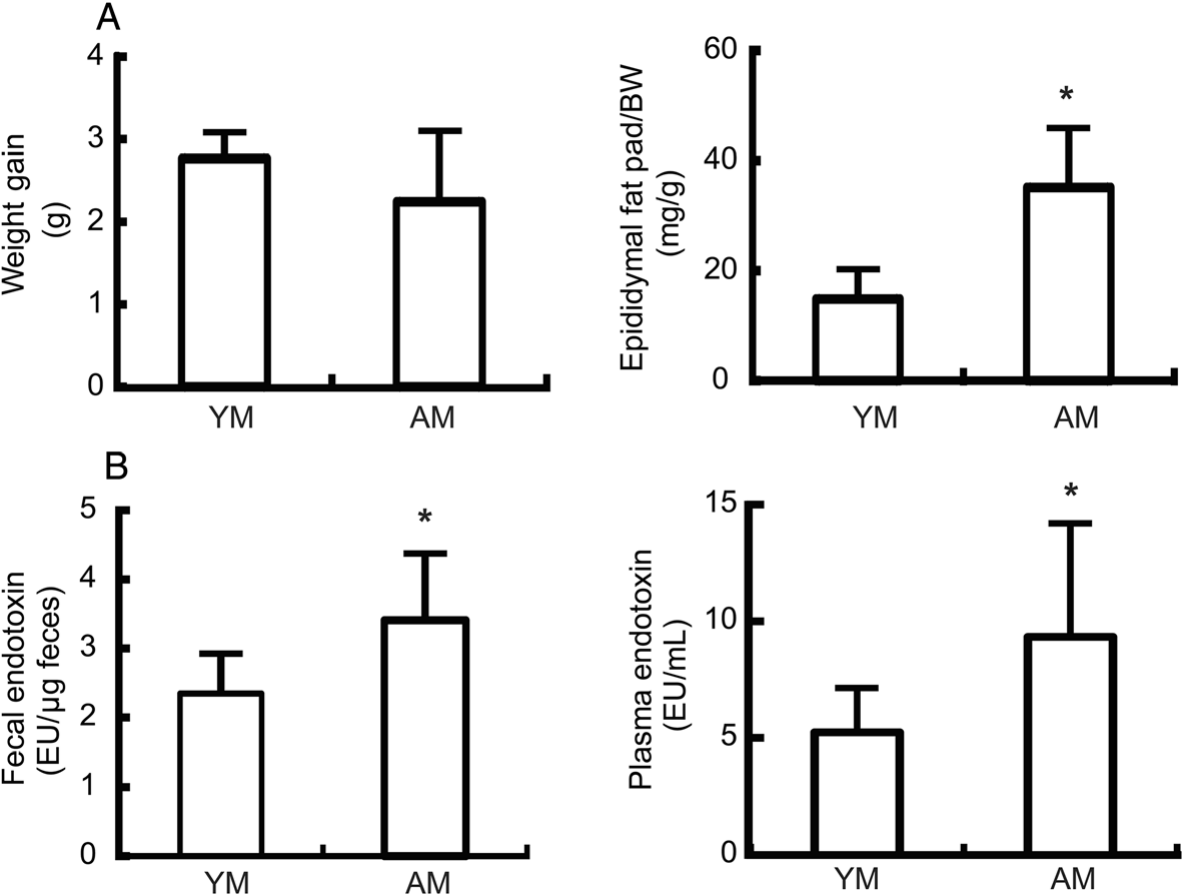
Effects of aging on endotoxin levels in young and aged mice. **a** Body weight (g) and epididymal fat pad weight (mg/g of body weight) of male C57BL/6 J mice (4 and 18 months old) were measured. **b** The fecal endotoxin concentration per gram (EU/g of feces) and plasma endotoxin concentration per mL (EU/mL) were measured using the *Limulus* amebocyte lysate assay. All values are indicated as the mean ± standard error of the mean (*n* = 8). *, *p* < 0.05 in comparison with young mice. YM, young mice; AM, aged mice

To understand the role of LPS on the aging in mice, we measured the LPS levels in young and aged mice. We detected a significant increase (*p* < 0.05) in the epididymal fat pad weight/body weight ratio in aged mice as compared to young mice although there were no significant differences in the body weight gain (Fig. 2a).

Next, we evaluated the endotoxin levels in the different treatment groups to investigate whether changes in gut microbiota composition with age are correlated with systemic endotoxemia. As shown in Fig. 2b, the fecal endotoxin levels in aged mice were higher than those in young mice. We also detected significantly higher systemic endotoxin levels in aged mice as compared to young mice.

### Expression levels of p16, cell cycle regulators, and SAMHD1 in young and aged mice

**Fig. 3:**
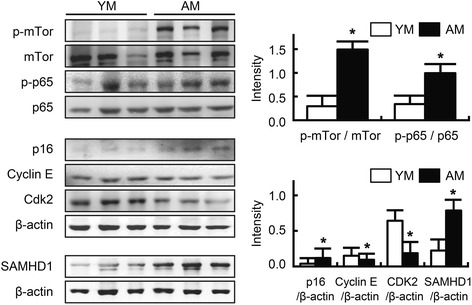
Effects of aging on inflammation, p16, cell cycle-regulators, and SAMHD1 levels in the colon of young and aged mice. Western blot analysis was performed on colon lysates from young mice or aged mice. All values are indicated as the mean ± standard error of the mean (*n* = 8). YM, young mice; AM, aged mice

Next, we measured the levels of p16, a senescence marker [6, 7], and cyclin E and CDK2, the G1/S-specific cell cycle regulators [18]. As indicated in Fig. 3, the expression of p16 was higher, while the expression levels of cyclin E and CDK2 were suppressed in aged mice relative to that in young mice. We also observed that activation of NF-κB and mTOR were stronger in aged mice than in young mice. Interestingly, concurrent with the increased expression of p16, the colonic expression of SAMHD1 was increased in aged mice relative to that in young mice.

**Fig. 4:**
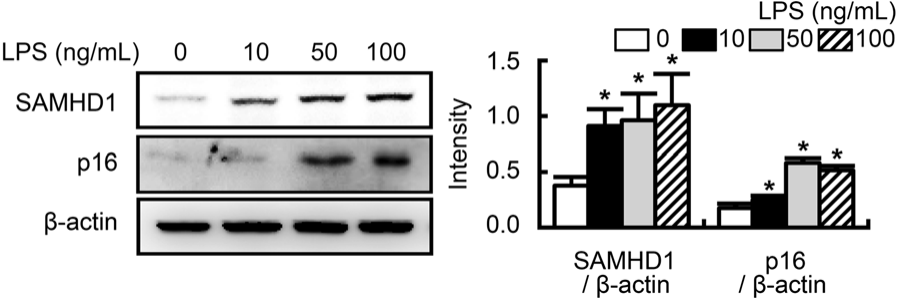
Effects of LPS on SAMHD1 expression in peritoneal macrophages. Peritoneal macrophages from mice were incubated with 10, 50, or 100 ng/mL lipopolysaccharides and used for immunoblotting. All values are indicated as the mean ± standard error of the mean (*n* = 3). YM, young mice; AM, aged mice

To investigate whether higher levels of lipopolysaccharide (LPS) may trigger increased expression of p16 and SAMHD1, we treated peritoneal macrophages isolated from young mice with LPS *ex vivo*. We found that LPS increased the expression of p16 and SAMHD1 (Fig. 4).

### LFL increased SAMHD1 expression and inflammation in isolated macrophages

**Fig. 5:**
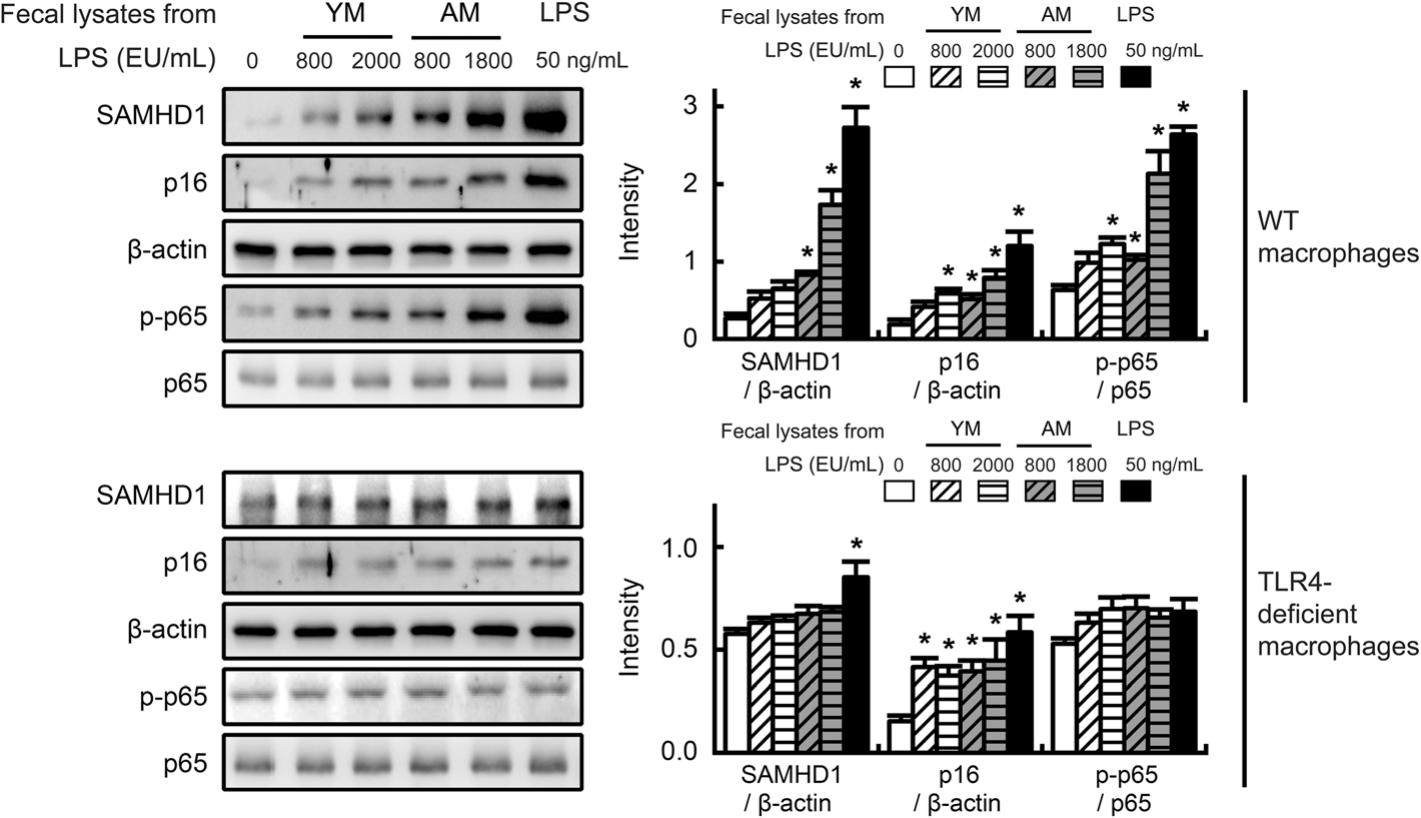
Effects of fecal lysates from young or aged mice on the expression levels of SAMHD1 and the senescence marker p16. Peritoneal macrophages from wild-type mice (top panel) and toll-like receptor 4-deficient mice (bottom panel) were incubated with fecal lysates from young or aged mice maintained on a low-fat or high-fat diet and then used for immunoblotting. All values are indicated as the mean ± standard error of the mean (*n* = 3). YM, young mice; AM, aged mice

To investigate whether intestinal bacterial endotoxin could induce p16 and SAMHD1 expression, we isolated peritoneal macrophages from wild-type (WT) and TLR4-deficient mice and incubated them with fecal lysates from young and aged mice. The LFLs from aged mice induced greater p16 and SAMHD1 expression in peritoneal macrophages from WT mice than did those from young mice when the isolated peritoneal macrophages were treated with different concentrations of LPS (Fig. 5 top). However, when peritoneal macrophages from TLR4-deficient mice were incubated with LFLs, they slightly but not significantly increased NF-κB activation and SAMHD1 expression, whereas they significantly induced p16 expression (Fig. 5 bottom). Nevertheless, p16 expression was induced more potently in macrophages from WT mice than in macrophages from TLR4-deficient mice. Moreover, the difference of p16 and SAMHD1 expression NF-κB activation between young and aged mice was not significant.

## Discussion

The human body inevitably fails due to aging. Although aging is not generally considered a disease unto itself, it facilitates inflammation, which may trigger aggressive degenerative diseases [19, 20]. Moreover, continued low-grade inflammation typical of aging, called “inflamm-aging” [21], may be the result of age-related changes in the community composition of the gut microbiota [3]. Decreased levels of *Bifidobacteria*, which has been shown to exhibit anti-inflammatory effects in mice, and increased levels of *Lactobacilli* and *Enterococci* have been reported in elderly individuals in comparison with young adults [4]. Hopkins et al. [22] reported that both *Bifidobacteria* and *Lactobacilli* levels were lower in elderly individuals than in young adults, whereas there were no differences in *Bacteroides* and *Eubacterium* levels. Moreover, the composition of the gut microbiota in the elderly is extremely variable between individulals [23, 24]. Others have reported higher levels of *Ruminococcus* and *Bifidobacteria* and lower levels of *Bacteroides* and *Eubacterium* in the elderly than in their younger counterparts [25, 26].

In this study, aging resulted in an increase in the phyla *Firmicutes* and *Actinobacteria* and a reduction in *Bacteroidetes* and *Tenericutes*, leading to an increase in the *Firmicutes* to *Bacteroidetes* ratio in the gut microbiota. Recently, Mariat et al. [27] reported that the *Firmicutes* to *Bacteroidetes* ratio evolves during different life stages in humans, with measured ratios of 0.4, 10.9, and 0.6 in infants, adults, and the elderly, respectively. These results suggest that the aging process may modify the composition of the gut microbiota in both animals and humans.

Recent studies have provided evidence that the gut microbiota may play an important role in the aging process, and imbalances between the pro-inflammatory and anti-inflammatory networks in the gut microbiota may result in low-grade chronic inflammation [28–30]. Additionally, aging induces the production of gut microbiota LPS in animals and humans [28–30]. Moreover, inflammatory markers such as LPS-binding proteins increase with advancing ages in healthy persons [17]. These support that the gut microbiota LPS facilitates low grade inflammatory status [30, 31].

In this study, we also found that fecal and systemic endotoxin levels were higher in aged mice than in young mice. Furthermore, NF-κB activation and p16 expression were stronger in the colon of aged mice. These results indicated that advancing age could disrupt gut microbiota, increase gut microbiota LPS production, cause intestinal inflammation, increase the intestinal permeability of LPS, and then generate and/or accelerate systemic endotoxemia and inflammation. Moreover, Stehle et al*.* [17] reported that inflammatory markers such as LPS-binding proteins in the blood of healthy persons increase with advancing ages. These results are supported by the previous reports that gut microbiota-induced endotoxemia is coupled with increased NF-κB activation [30, 31].

The stimulation of LPS induces SAMHD1 expression in the macrophages [32]. SAMHD1 regulates cell proliferation by depleting the cellular dNTP pool [12, 13]. In the present study, we also found that commercial LPS and LFLs, the LPS fraction of gut microbiota of mice, induced a much greater increase in SAMHD1 and p16 expression in the isolated peritoneal macrophages from WT mice. SAMHD1 and p16 expression was induced more potently by the LFL from aged mice than by that from young mice. These results suggested that aging could induce the production of gut microbiota LPS production and the LPS produced by the gut microbiota from aged mice induce SAMHD1 and p16 expression more potent than that from young mice. However, when the peritoneal macrophages from TLR4-deficient mice were stimulated with the LFLs from young and aged mice, SAMHD1 expression and NF-κB activation slightly but not significantly induced by the LFLs. However, the LFLs induced the expression of p16, which was weaker in the macrophages from wild-type than in the macrophages from TLR4-dificent mice. Moreover, the induction of p16 expression was different between the LFLs from young and aged mice. Additionally, Stehle et al*.* [17] reported that LPS-binding proteins such as CD14 in the blood of healthy persons increase with advancing ages. These results suggest that the SAMHD1 and p16 expression may be LPS-inducible through the LPS-TLR4-dependent signal pathway.

Furthermore, we found that the expression of cyclin E and CDK2, G_1_/S-specific regulators, decreased in aged mice more than in young mice. Recently, Franzolin et al. [33] reported that SAMHD1 is a major regulator of DNA precursor pools in mammalian cells and is variably expressed during the cell cycle, maximally during quiescence, and minimally during the S-phase. Furthermore, St Gelais et al*.* [34] also reported several SAMHD1-interacting cellular proteins such as cyclin A2, cyclin B1, CDK1, and CDK2 to play an important role in HIV-1 restriction function. These results suggest that age-induced induction of SAMHD1 may be associated with cell cycle regulation by modulating the levels of G_1_/S-specific regulators.

## Conclusion

These results indicated that aging could accelerate inflamm-aging by inducing p16 and SAMHD1 expression and NF-κB activation in mice via increasing gut microbiota LPS levels. In the present study, we demonstrated that SAMHD1 expression increased in aged mice burdened with inflammation, or an inflamm-aging condition, suggesting that SAMHD1 could serve as a novel marker of inflamm-aging in mice.
